# Development of a survey tool to assess emotional and social behavioral competencies of science technology engineering math and medicine (STEMM) graduate students

**DOI:** 10.1371/journal.pone.0328308

**Published:** 2025-09-02

**Authors:** Joann Farrell Quinn, Philip A. Cola, Melissa Cooper, Casey Miller, on behalf of University of South Florida Muma College of Business

**Affiliations:** 1 University of South Florida Morsani College of Medicine, Tampa, Florida, United States of America; 2 Department of Design and Innovation, Weatherhead School of Management, Case Western Reserve University, Cleveland, Ohio, United States of America; 3 Department of Population and Quantitative Health Sciences, Case Western Reserve University, Cleveland, Ohio, United States of America; 4 Department of Organizational Behavior, Weatherhead School of Management, Case Western Reserve University, Cleveland, Ohio, United States of America; 5 College of Science, Rochester Institute of Technology, Rochester, New York, United States of America; Tecnologico de Monterrey, MEXICO

## Abstract

The underrepresentation of women and minority students in STEMM graduate programs remain a significant challenge, compounded by biases in traditional admissions processes and barriers to effective mentoring and retention. This study develops and validates the Quinn Miller Competency Assessment (QMCA), a tool designed to assess emotional and social intelligence (ESI) competencies crucial for STEMM graduate student success. The QMCA was created through an iterative process involving literature review, expert consultations, and empirical studies. It evaluates five key competencies: self-awareness, self-control, adaptability, achievement orientation, and teamwork. The tool’s validity and reliability were tested using exploratory and confirmatory factor analyses on diverse samples of STEMM graduate students and applicants. Results demonstrated strong construct validity and reliability and invariance across gender and race/ethnicity supporting the QMCA’s use in both admissions and student development contexts, in conjunction with other measures. By enabling a more holistic evaluation of applicants’ competencies through an assessment that fairly and consistently evaluates individuals across demographic groups, the QMCA aims to improve access and retention for underrepresented groups in STEMM fields, fostering a more inclusive and diverse scientific community. Future research will specifically explore how the QMCA can enhance holistic evaluation processes and contribute to student retention and success efforts. Additionally, we will test its applicability across broader disciplines.

## Introduction

The access and retention of US women and underrepresented minorities in science, technology, engineering, mathematics and medicine (STEMM) PhD programs continue to be problematic in higher education [1,2]. It is well documented that the traditional admissions process in graduate education selects against women and underrepresented minorities (URMs = African American, Hispanic American, and Native American) [1,3]. Biases and barriers can also manifest after admission resulting in disparities in access to effective mentoring, support systems, retention and completion [3,4]. Addressing these issues is not only critical for individual graduate student success, but also for the overall diversity and vitality of academia. When institutions neglect to foster an inclusive environment, they risk driving away talented individuals, thereby depleting the pool of diverse approaches, perspectives, and voices necessary for cutting edge research and development [3,5]. To address these issues, the National Science Foundation INCLUDES Alliance: IGEN (Inclusive Graduate Education Network [cite: https://igenetwork.org/]) is a cross-sector collaboration aimed at improving access and retention in for underrepresented racial and ethnic minority (UREM) students in in STEM PhD programs, particularly in the physical sciences. With participation from over 30 organizations, IGEN promotes inclusive, evidence-based practices for graduate admissions and mentoring. To enhance access, IGEN helped establish Bridge-to-the-Doctorate programs to help students overlooked by traditional admissions processes, while also training faculty on holistic admissions practices [6] To improve retention, IGEN provides evidence-based support structures for graduate students, such as equity-minded mentoring workshops and emotional intelligence training for faculty staff and students.

A key strategy in advancing IGEN’s mission is the development of assessment tools that impartially evaluate students’ emotional and social intelligence (ESI) competencies, which are critical for success in STEM. Research indicates that higher levels of emotional intelligence is associated with better academic performance, psychological well-being, and resilience among graduate students. A study with university students found that emotional intelligence positively correlates with self-efficacy, motivation, and resilience, which are factors that enhance both psychological well-being and academic achievement. These effects were particularly pronounced among postgraduate students, highlighting the importance of emotional intelligence in advanced academic settings. [7].

From their experience in building Fisk-Vanderbilt’s renowned Bridge program [8]. Stassun et al. argue that one key to promoting diversity in physics graduate education is to identify and then cultivate “unrealized or unrecognized potential” held by individuals often overlooked by the typical admissions and recruitment system. ESI competencies serve as indicators of such unrealized potential, as prior research suggests that such non-cognitive factors play a role in academic success and persistence in graduate education. As the world and work becomes more collaborative and interconnected, ESI competencies rise in importance as foundational abilities critical for personal, professional, and societal success, ensuring individuals can effectively navigate complex social networks and diverse environments.

Measures of ESI may be well suited to identifying unrealized potential in individuals historically marginalized in STEMM, as ESI measures have been demonstrated to be invariant across race and gender [9–11]. Although reliable, valid, and systematic assessments of emotional intelligence constructs may be critical in this regard, ESI research has historically neglected graduate education in STEMM fields [12] and systematic assessments of ESI competencies directly addressing such for STEMM programs have been lacking, limiting the ability of programs to integrate these attributes into admissions and student success efforts.

This study introduces the Quinn Miller Competency Assessment (QMCA), a newly developed tool designed to assess five key ESI competencies (i.e., self-awareness, self-control, adaptability, achievement orientation, and teamwork) in STEMM graduate students and graduate applicants. While ESI have been linked to student outcomes in broader educational settings, the primary objective of this research is to validate the QMCA as a reliable and psychometrically sound assessment tool, ensuring that it effectively and impartially measures these competencies for future use in both admissions and mentoring contexts for STEMM program success. We anticipate that future research will explore how the QMCA can enhance holistic evaluation processes and contribute to student retention and success efforts.

### Background and literature review

ESI has been a hot topic for the last few decades and continues to be a topic of increasing interest across cultures, sectors, disciplines, etc. [13] with emotional and social competencies becoming increasingly important in the post-COVID-19 era [14] Prior research supports the importance of ESI in performance [15,16]and well-being [17,18]. While studies in ESI are numerous, the bulk of the research has been in traditional business environments, not in areas such as the natural sciences, and even less of a focus has been within higher education. However, success in higher education demands not only cognitive capabilities but also emotional resilience and the ability to adeptly manage social interactions. ESI are crucial as they shape students’ experiences, educator-student dynamics, and collaborative research endeavors. Natural sciences can be particularly challenging due to the intrinsic complexity of content and research demands. Emotional competencies refer to the ability to recognize, understand, and manage one’s own emotions and the emotions of others. These competencies are crucial for effective self-regulation, resilience, and interpersonal interactions. Key emotional and social competencies include a framework that comprises self-awareness, self-management, social awareness, and relationship management.

### Relevance in STEMM graduate education

The inclusion of ESI in STEMM graduate education is particularly important due to the collaborative and interdisciplinary nature of scientific research. Success in STEMM fields often requires not only technical expertise but also the ability to work effectively in teams, communicate complex ideas, and navigate interpersonal dynamics [19].

By assessing and developing ESI, educational institutions can better prepare STEMM graduate students for the multifaceted challenges of their future careers. This holistic approach to education can enhance not only individual student outcomes but also the overall diversity and innovation within the scientific community [2] Below we briefly review how assessing ESI supports student admissions and recruitment and retention and student success. Next, we more closely examine three categories of ESI in relation to STEMM graduate student success: self-awareness, self-management and relationship management. Finally, we explore how ESI assessments can improve access and retention for women and underserved minorities in STEMM Programs.

### Admission and recruitment

ESI competencies are widely recognized as critical factors influencing personal and professional success. Research in organizational behavior and psychology suggests that competencies such as self-awareness, adaptability, and teamwork contribute to leadership effectiveness, problem-solving, and resilience [20]. In higher education, particularly in professional fields such as medicine and law, non-cognitive assessments have been explored as a supplement to traditional academic metrics [21] previously.

In the STEMM context, emotional and social competencies may likely play a particularly important role given the highly collaborative nature of scientific research and the persistence and adaptability required to complete challenging graduate programs. Prior studies indicate that social support networks, mentorship quality, and self-regulation skills are associated with student retention in STEMM PhD programs [2,22]. However, research explicitly linking emotional intelligence competencies to graduate student success in STEMM remains limited. The QMCA scale developed in this study is intended as a first step toward addressing this gap by providing a validated instrument for assessing ESI competencies in STEMM graduate students.

While some studies suggest that holistic admissions, including assessments of ESI competencies, may help identify students with strong potential for success beyond cognitive metrics alone, the QMCA’s specific impact on admissions outcomes or minority student retention has yet to be tested. Future studies should examine whether incorporating the QMCA into graduate admissions or mentoring programs influences the selection and success of underrepresented students in STEMM.

### Retention and student success

Once a student is admitted, challenges to success do not end. The literature discusses the challenges faced by minority students, including feelings of isolation and lack of belonging [22]. These authors argued that social skills, such as networking and building relationships with peers and mentors, are essential for creating a supportive learning environment, which in turn aids retention. Tinto posits in his theory of student departure that a lack of social integration is a key factor leading to student dropout [23]. Minority students often struggle with cultural and social integration into predominantly white institutions, which can lead to feelings of isolation and alienation. Others further elaborate that these students frequently face difficulties in assimilating into the college environment, which can impede their academic and social integration [24].

Coaching and mentoring are widely accepted methods for supporting student retention and success. Coaching and mentoring, though often used interchangeably, are distinct concepts with unique characteristics and objectives. According to Grant, coaching is a collaborative, solution-focused, result-oriented, and systematic process in which the coach facilitates the enhancement of work performance, life experience, self-directed learning, and personal growth of the coachee [25]. Mentoring is a more long-term relationship where a more experienced individual (the mentor) provides guidance, advice, and support to a less experienced person (the mentee). Kram in her seminal work “Mentoring at Work” describes mentoring as a relationship that involves career and psychosocial support [26]. The mentor’s role is not only to guide in professional matters but also to assist in the personal growth of the mentee. Both coaching and mentoring may support student retention and success by focusing on the development of ESI competencies, with coaching following a prescribed plan for supporting the individual in their own development, while mentors can offer role modeling, as well as advice and the sharing of experiences. In turn, improved ESI competencies can enhance mentoring relationships that support student success.

### Emotional and social competencies

In terms of emotional intelligence, there is evidence that the competencies of self-awareness (knowing ones’ emotional states, preferences, resources, and intuitions) and self-management (managing ones’ internal states, impulses, and resources) may be of importance for graduate students in STEMM [27] In particular, emotional skills such as resilience and adaptability are essential when facing research hurdles. As posited nearly a decade ago researchers found that emotional competencies directly influence a student’s research motivation and persistence [28]. There are two broad categories of emotional intelligence: self-awareness and self-management.

Self-awareness broadly refers to an individual’s ability to introspect and recognize their own thoughts, emotions, strengths, weaknesses, and values [29]. In particular, awareness of one’s emotions and their effects serve as a foundation for other aspects of emotional and social intelligence. By recognizing emotional triggers and patterns, individuals gain better control over their reactions, fostering self-control and the ability to adapt to new circumstances. Research shows that leaders with higher self-awareness can decipher their emotional responses and understand how their emotions impact their behavior making them better equipped to navigate complex organizational landscapes [30]. Self-awareness fosters improved interpersonal relationships through the preemptive management of potential emotional conflicts [31]. which enhances collaboration and overall teamwork. Research has even indicated that developing emotional intelligence, which includes self-awareness, can positively impact students’ achievement in STEM disciplines [32].

While self-awareness serves as a foundation, self-management is the capability that builds on this awareness, allowing individuals to regulate their emotional reactions [31,33]. Self-management refers to an individual’s capacity to actively regulate their emotions, behaviors, and commitments to achieve desired goals.

Evaluating self-management skills such as adaptability, self-control, and achievement orientation can aid in identifying candidates who possess the necessary discipline and perseverance required for success in demanding graduate programs and PhD-level roles in the STEMM workforce. Adaptability, or being able to adjust to new conditions is essential for those involved in research, as is self-control (being able to respond with thoughtful behavior instead of reacting without thinking), and achievement orientation keeps an individual motivated to keep moving forward. Assessing self-management for terminal degree programs can provide valuable insights into an applicant’s ability to effectively manage themselves [34], including their time, resources, and personal well-being. Self-management skills support the individual in navigating and excelling in demanding and self-directed terminal degree programs.

Scientific endeavors often involve long hours of experimentation, data collection, and analysis. These processes can be tedious and sometimes yield unexpected or unwanted results. Self-control enables scientists to maintain focus, avoid premature conclusions, and remain patient throughout their investigations; scientific misconduct can arguably be traced back to deficits in self-control. Furthermore, it helps in persevering through the inevitable setbacks and obstacles that arise during research. Adaptability allows scientists to be flexible in their approaches, adjusting their methodologies or hypotheses considering new data or technologies. An achievement orientation ensures that scientists are motivated to excel, set high standards for their work, and are committed to making a meaningful impact in their field. It drives them to push the boundaries of what’s known, take calculated risks, and persistently seek answers.

### Relationship management

Relationship management refers to the ability to build and maintain positive relationships with others, including effective communication, collaboration, and conflict resolution. The ability to work collaboratively allows individuals to pool their collective knowledge, skills, and resources, often leading to enhanced outcomes and innovations. Recent research has referred to this as a relational capacity, which is the ability to develop deep and meaningful bi-directional relationships that allow for improved performance [35]. Every role has an element of teamwork. This is particularly true for research in natural sciences, which often involves students and faculty working together in highly interdependent lab environments.

Teamwork in particular may be key to successful STEMM graduate study. A key social competency, teamwork involves effective communication, understanding, and capitalizing on diverse viewpoints, and leveraging the collective strengths of individuals. Scholars have emphasized that students and researchers with such social competencies can foster a collaborative environment, ensuring research efficacy [36]. In addition, these social skills allow students to effectively communicate with educators, enhancing the quality of their interactions which in turn can significantly affect learning outcomes [37]. Being an effective team member is no longer just a commendable skill but a crucial competency. Working in teams provides a support system for members that is crucial during challenging phases of a project, helps foster resilience, and aids collective problem-solving [38].

### Use of ESI assessments in student selection and development

While the body of work devoted to studying the validity of non-cognitive constructs in selection is enormous in organizational studies, studies specifically in higher education and particularly STEMM PhD programs are few. However, non-cognitive assessments have been used successfully in dental school admissions [27,39,40] and were shown to have validity beyond cognitive measures for long term performance of medical students^.^ Lievens et al. showed that the correlation of non-cognitive competencies with performance in medical school [41] and long-term success [21] increased with time, while the correlation with cognitive tests decreased with time. Similar results from a study of lawyering effectiveness have been used to suggest that non-cognitive competencies be used to transform law school admissions [38]. In addition to being meaningful predictors of performance, measures of emotional intelligence are useful tools to increase access to underrepresented groups in STEMM because they do not show major group differences based on gender or race [9–11]. Indeed, research suggests that non-cognitive assessments in areas of emotional intelligence, self-efficacy, outcome expectations and interest can specifically support access and retention of underrepresented minorities in STEMM PhD programs [42,43].

In addition to enhancing the selection process, ESI assessments may also be used to support graduate student development and retention. Competency-based ESI assessments focus on assessing behaviors as opposed to individual traits, and as such, have a long history of use supporting individual development. By assessing themselves and reviewing the results of other’s evaluations of their competencies, individuals gain insights into their strengths and areas for improvement, fostering deeper self-awareness and facilitates personal growth [44]. Thus, assessments may be used in mentoring and support programs to help STEMM graduate students further develop these core ESI competencies. Using the assessment to support ESI development can in turn foster increased retention. One study underscored that students with refined emotional competencies can better handle academic pressures, leading to improved well-being and retention rate^s^ [45]. As members of underrepresented groups may encounter increased challenges and stress during college and thus would particularly benefit from tools that support the development of their emotional and social competencies.

### Need for new ESI assessment

Although we initially sought to employ an existing assessment of ESI, we found that none were designed for purposes of both selection and development in a STEMM education context. Those intended to support personal growth and development, such as the Emotional and Social Competency Inventory (ESCI) [46] are often designed for holistic development, and are widely used in organizations; however, this tool has been developed and tested with the target population, and only includes a select few competencies deemed to be most important for success in higher education. In addition, as prediction is not a primary goal of developmental assessments, they are often used without controlling for method bias in self-ratings. When assessments are used in selection processes, social desirability significantly impact self-report ratings [47], making inclusion to control this bias more crucial. As our aim was to also use the assessment to help students develop their competencies, we adopt a behavioral approach to social emotional intelligence, focusing on assessing behavioral competencies that can be developed. This ruled out use of trait measures of emotional intelligence [48]. This led us to create our own assessment tool, the Quinn Miller Competency Assessment (QMCA). This strategic shift allowed us to address the gap in selection-focused assessments while recognizing the critical importance of incorporating a discrete set of competencies valued by the STEMM community. After many rounds of refinement, the QMCA includes a set of ESI competencies with the intention of the tool to address self-development.

## Materials and methods

In a series of studies, we identify a set of ESI competencies related to STEMM student success and develop the QMCA tool to assess the competencies. Establishing scale validity is essential prior to scale use, as it ensures that the data collected is accurate and meaningful, and that survey items consistently measure targeted concepts. We used an iterative process of empirical and conceptual analysis to inform the development of the QMCA. Stage 1 informed selection of specific ESI constructs and item creation. At Stage 2 we conducted two studies to reduce the number of ESI constructs and inform item development (Studies A & B). At Stage 3, we confirmed the measurement model and assessed construct validity and invariance (Study C). Table 1 includes demographic information for each sample. To address concerns about socially desirable responding in evaluation of self-report responses, a particular concern for measures used in selection processes, the research team concurrently tested the QMCA with various social desirability controls during stages 2 and 3.

**Table 1:** Sample Demographics.

Study	Sample Size	Pool	Gender (male)^1^	Race, Ethnicity, Nationality^1^	
Study A	467	Applicants, students & graduates of physics graduate programs from 275 global colleges & universities in 2019	61.9%	Asian or Asian American Black or African American Hispanic or Latinx White or Caucasian Non-US citizen Other Not Disclosed	17.9% 5.0% 13.7% 59.7% 0.7% 2.0% 1.0%
Study B	354	Students & recent graduates of STEMM PhD programs from 15 U.S. colleges & universities in 2020	59.3%	Asian American Black or African American Hispanic or Latino/Latina White or Caucasian American Non-US citizen Other Not Disclosed	7.9% 2.8% 4.5% 43.8% 35.3% 0.3% 5.4%
Study C	330	Applicants to STEMM graduate programs in 2022	23.6%	Asian or Asian American Black or African American Hispanic or Latinx White or Caucasian Other Not Disclosed	11.2% 13.0% 13.9% 57.9% 1.8% 2.1%

^1^Percentages excluding missing cases.

The QMCA, like other measures of behavioral competencies, provides individual scores by competency, it is not a single score assessment that provides an overall measure of emotional and social intelligence. While emotional and social competencies are broadly deemed to be interconnected, measuring competencies individually enables insights into how an individual may excel in one competency, but require development in another, providing pathways for targeted developmental opportunities. The final scale together with instructions on how to score the scale are included in S1 Appendix.

This study was submitted to the Rochester Institute of Technology Institutional Review Board and approved as human subject research. All data collection and proceeding analyses were performed in accordance with relevant guidelines and regulations. Informed consent was provided within the survey used for data collection and was accepted by all participants within the sample. All identifying information, if provided, was removed prior to analysis.

**Competency Selection & Measure Development**. (**Stage 1).** We began the process by conducting a literature review and engaging a representative panel of experts, primarily composed of faculty and research scientists in physics and astronomy, to pinpoint the non-cognitive (emotional and social) competencies most pertinent to success in a STEMM PhD program. Two members of the research team then selected a final set of ESI competency constructs and created initial items. We then recruited a focus group of STEMM PhD program directors to assess the content validity of the ESI competencies and iterated on item development.

**Construct and Item reduction and refinement (Stage 2).** At stage 2, we assessed performance of the selected ESI competency measures in the target population of students engaged in or applying to STEMM graduate school programs and iterated on item development. In Study A, we assessed the factor structure of the ESI constructs in a sample of 467 applicants for the 2019–2020 academic year to nine physics and/or astronomy PhD programs at private and public institutions geographically spread across the US, as well as programs affiliated with the Southeastern Compact for Inclusive Student Transitions in Engineering and Physical Sciences (SCI-STEPS), and student fellows associated with the American Physical Society’s Bridge Program. Students were matriculated at 275 colleges and universities around the world. Based on our empirical findings and additional theoretical review, we revised the set of ESI competencies and their measures and tested them in Winter 2020 in a student sample of 364 STEMM PhD students at large public research institution in the southeast (Study B). Students in Study B represented a wider range of STEMM disciplines (Physics: 24%, Engineering: 20.3%, Chemistry: 17.8%, Computer Science: 13.8%. Mathematics: 12.7%, Earth Sciences: 10.5%). In Study C, we re-tested factor structure and reduced redundancy and improved item-factor alignment. Scale revision at Stage 2 was also informed by iterative reviews of construct definitions for the included social and emotional intelligence^50^ and Grit competencies^51^ and measures of closely related competencies such as academic achievement motivation^51^ and career related adaptability^52^.

**Scale validation (Stage 3).** In Study C, we tested the final QMCA in a representative sample of 330 applicants for 2022−23 STEMM graduate programs, our focal population, to validate construct validity and reliability of each factor measure and to conduct preliminary tests of invariance across sex and race/ethnicity. The final QMCA is presented in S1 Appendix.

## Results and discussion

### Stage 1. Competency Selection & Measure development

Faculty and researchers at the 2017 Joint Graduate Education and Bridge Program Conference of the American Physical Society and the American Astronomical Society’s Women in Astronomy IV Conference (2017) were asked to identify non-cognitive competencies valuable for STEMM graduate studies. The two groups agreed that the same clusters of non-cognitive competencies are important for research success: self-management (e.g., Optimism, Trustworthiness, Achievement Orientation, Conscientiousness, Adaptability, and Initiative) and self-awareness (e.g., Accurate Self-Assessment, Self-Confidence). The initial version of the QMCA was constructed based on these results, adding two competencies identified through literature review (i.e., professionalism and grit). Two members of the research team developed items for each measure based on a behavioral competency perspective. The literature was reviewed for behavior-based emotional and social competencies, with reference to the item structure employed in the Emotional and Social Competency Inventory (ESCI), which is a well-validated instrument used in many countries. Once items were created based upon the literature and understanding of psychometric properties, expert/user focus groups and interviews were used to assess the competency constructs and their items. Following this stage, both expert and non-expert review continued via content validity surveys. While the final competencies selected for inclusion were based on extensive field testing with physicists and astronomers, the assessment was created in a manner that is generalizable so that it may be utilized throughout STEMM, or at least the physical sciences.

Stage 1 resulted in the development of a survey that measured seven ESI competencies: Self-awareness (6 items), Self-control (5 items), Professionalism (7 items), Teamwork (7 items), Achievement Orientation (7 items), Adaptability (6 items) and Grit (8 items) and a 4-item measure commonly used to control for Social Desirability bias in student populations [49]. Except for the Grit^53^ measure all items were newly generated. All Items were rated on a five-point Likert Scale (1 = Never to 5 = Consistently). To enable us to screen for accessibility of the items, participants were also given an option to select “I don’t know”.

### Stage 2. Scale Reduction and Revision

**Study A.** Our initial screening for item clarity found no problematic items. Out of the 50 items, only 6 received a single “I don’t know” response, with two coming from the same respondent. Next, we used exploratory factor analysis (EFA) to assess factor structure and construct redundancy. An initial 13-factor solution was recommended from principal components analysis with Promax rotation based on eigen values (1.05) and the scree plot. We further explored factor structure by constraining the model to 8 factors (reflecting the 7 ESI competencies and social desirability) using Maximum Likelihood extraction with Promax rotation. After an iterative process involving removal of items that failed to load or that significantly cross-loaded, 20 items, including all the social desirability items, were removed from the scale. The remaining 30 items revealed an adequate 7-factor model (KMO = .816, Bartlett’s Test of Sphericity < .05), which explained 38.8% of the variance. However, communalities for each factor were barely sufficient (<.20) and factor scales failed to demonstrate adequate reliability (α’s < .70).

We revised the scale based on the conceptual objectives of each factor measure and our empirical results. Professionalism, Grit, and Social Desirability performed poorly, with items either failing to load or more than half the items cross loading. Professionalism was excluded because only those items related to timeliness loaded on an independent factor. EFA results also indicated redundancy between Grit and Achievement Orientation, however items from the “perseverance of effort” dimension of Grit loaded independently. Thus, we replaced Grit with Perseverance, defined as persisting in a course of action and finishing what one starts [50] measuring it with 6 items from the original Grit scale [51]. The academic based social desirability scale performed poorly in this sample, possibly due to its focus on math engagement and performance, thus we used a more generic social desirability measure [52] in the next data collection. Finally, we reviewed and revised items that cross-loaded to increase their correspondence to the construct definition of their referent factor.

Beyond the conceptual review we assessed potential methodological threats to the scale. The initial QMCA mixed negatively and positively worded items to reduce response bias, however, recent research has shown that this approach can be ineffective and including negatively worded items can rather threaten validity and reliability of scales [53,54]. In fact, in our sample, nearly half of the negatively worded items failed to load properly and removing these items increased the explained variance from 38.8% to 46.4%. Based on this, we positively worded all items in the next scale iteration. The revised QMCA assessed six emotional and social competencies: self-awareness (5 items), self-control (5 items), teamwork (5 items), achievement orientation (5 items), adaptability (5 items) and perseverance (6 items [52]) and included a 5-item social desirability measure [55]. All scales were rated on a 5-point Likert scale (1 = Never to 5 = Consistently).

**Study B.** Principal Components analysis yielded a 7-factor model based on eigen values (1.12) and the scree plot when the QMCA was tested with the social desirability control. However, perseverance split across two factors and self-control items failed to load on a single factor. The social desirability scale again failed to load on a unique factor and was omitted from the further analysis. We used EFA analysis with Maximum Likelihood extraction and Promax rotation constraining the model to 6 factors to explore the factor structure. EFAs revealed that 3 of the 6 Perseverance items loaded onto the Achievement Orientation factor (β range of cross-loaded items:.51 to.83). We removed perseverance from the QMCA because it failed to load on a unique factor. This decision was supported by the relatively small reduction in variance explained when Perseverance was omitted (6-factor model = 43.55%, 5-factor model = 41.99%).

The 5-factor EFA with Maximum Likelihood extraction and Promax rotation performed well with all but 2 items loading to their intended constructs (β range:.41 to.80) without significant cross loadings (β range of cross-loaded items: −.26 to.27; see Table 2 for all loadings and cross-loadings in the final full 5-factor model). After removing one self-control item that failed to load on any factor, the resulting model (24 items) performed well (KMO = .874, Bartlett’s Test of Sphericity < .001), with each factor measure demonstrating adequate reliability (α range:.71−.78), and the model explained 42.84% of the variance in the sample, however communalities continued to be predominantly low (20 items < .50; 4 items between.50 and.60). Based on these results, we reviewed and revised item text to increase their convergence around each target factor definition. This produced a final 25 item tool assessing 5 competencies: self-awareness, self-control, adaptability, achievement orientation and teamwork. The full text of the final measures are in S1 Appendix together with instructions on calculating factor scores or using average values, calculating the self-deception variable as well as how to use the method controls.

**Table 2:** Exploratory Factory Analysis (EFA) Factor Loadings.

Dimension	Item	Factor Loadings				
		SA	SC	AD	AO	TM
Self-Awareness (SA)	SA1	.52				
	SA2	.80				
	SA3	.61				
	SA4	.70	−.20			
	SA5	.41				
Self-Control (SC)	SC1		.59			
	SC2		.54	.21		
	SC3	.29	.17	.16		
	SC4		.55			
	SC5		.74			
Adaptability (AD)	AD1			.49		
	AD2			.69		
	AD3			.67		
	AD4			.69		
	AD5		.29	.34		.23
Achievement Orientation (AO)	AO1		.16		.74	
	AO2		−.26		.44	
	AO3				.63	
	AO4				.55	
	AO5				.67	
Teamwork (TM)	TM1			.16		.58
	TM2			.26		.54
	TM3			−.17		.66
	TM4					.76
	TM5					.57

Study B N = 354. Standardized loadings for the QMCA factors were estimated using exploratory factor analysis with a model constrained to 5 factors and using Maximum Likelihood estimation with Promax rotation.

As the initially selected social desirability measures failed to isolate method bias of STEMM students in Studies A and B, we conducted a comprehensive literature review to identify a new approach. Given that the QMCA requires a significant amount of introspection and is intended for use in stu dent selection and development, we follow Paulhus^’^ [55,56] recommendation and control for two types of social desirability bias: impression management and self-deception. Impression management involves consciously altering responses to create more favorable impression [57], which can be more prevalent when assessments are used in competitive selection processes [58]. Self-deception refers to positively biased responses that respondents believe to be accurate [56], a potential issue when assessing intangible traits like emotional and social competencies. We adapted an existing self-promotion measure to assess impression management. To assess self-deception, we created a new measure which targets two factors that lead to inaccurate self-evaluation of social-emotional competencies: [1] underestimating the impact of emotions on behavior and [2] overestimating one’s ability to assess their own skills or predict responses in various situations [52]. As self-deception involves denying flaws or exaggerating positive traits to an unrealistic extent, we structured the measure as the sum of a participant’s extreme responses on five items. The full text of the social desirability measures is included with the QMCA scale in S1 Appendix.

### Assessment tool evaluation: Discriminant and convergent validity and invariance (Stage 3)

#### Discriminant and convergent validity testing.

In Study C we used confirmatory factor analysis (CFA) in MPlus to assess the performance of the final version of the QMCA in assessing 5 ESI competencies deemed key to STEMM student performance (self-awareness, self-control, adaptability, achievement orientation and teamwork, 25 items total). We controlled for social desirability with a 5-item impression management factor and (5-item factor) and a self-deception variable (aggregate score of 5 items). The model fit the data well. Although chi-square was significant χ^2^(414)=785.706, p < .001, comparative fit measures were good and absolute measures demonstrated close fit [CFI = .92, RMSEA = .05, p-close = .26] based on recommended standards (CFI > .90, RMSEA < .06, p-close > .05, [59,60]).

Convergent and discriminant validity of the 5 ESI competency measures was supported in the STEMM student sample. Measures of each dimension demonstrated high reliability (Cronbach’s α’s:.74−.89, Composite Reliabilities:.75−.86). Table 3 reports all factor loadings. Factor loadings ranged from.50 to.85 exceeding the recommended thresholds for convergent validity (loadings > .4). Average variance extracted (AVE) was high for the adaptability, achievement orientation and teamwork competencies (AVE range:.55−.56) and marginal for the self-awareness and self-control competencies (AVE range = .38−.42). There was strong support for discriminant validity across the five dimensions with only moderate factor correlations (*R* range:.49−.67, see Table 4 for all factor correlations), and the AVE exceeded the maximum-shared variance (MSV) for each factor (***Δ***AVE-MSV range:.12 −.30). Table 5 reports results of all convergent and discriminant validity tests.

**Table 3:** Confirmatory Factory Analysis (CFA) Factor Loadings.

Dimension	Item	Factor Loadings (SE)				
Self-Awareness (SA)	SA1	.67 (.06)				
	SA2	.74 (.05)				
	SA3	.56 (.06)				
	SA4	.59 (.06)				
	SA5	.50 (.06)				
Self-Control (SC)	SC1		.63 (.05)			
	SC2		.76 (.05)			
	SC3		.62 (.05)			
	SC4		.67 (.05)			
	SC5		.54 (.06)			
Adaptability (AD)	AD1			.63 (.05)		
	AD2			.72 (.05)		
	AD3			.78 (.05)		
	AD4			.76 (.05)		
	AD5			.61 (.05)		
Achievement Orientation (AO)	AO1				.78 (.05)	
	AO2				.82 (.05)	
	AO3				.80 (.05)	
	AO4				.77 (.05)	
	AO5				.75 (.05)	
Teamwork (TM)	TM1					.62 (.05)
	TM2					.66 (.05)
	TM3					.66 (.05)
	TM4					.74 (.05)
	TM5					.77 (.05)

Study C N = 330. Standardized loadings for the QMCA factors were estimated using confirmatory factor analysis modeled with method factor controls (self-promotion and self-deception).

**Table 4:** QMCA Descriptive Statistics.

	Mean	SD	SA	SC	AD	AO	TM	SP	SD
**Self-Awareness (SA)**	4.07	.54	(.74)						
**Self- Control (SC)**	3.54	.62	0.39	(.78)					
**Adaptability (AD)**	3.80	.67	0.49	0.67	(.82)				
**Achievement Orientation (AO)**	4.31	.64	0.44	0.34	0.58	(.89)			
**Team (TM)**	3.80	.67	0.43	0.43	0.51	0.50	(.82)		
**Self-promotion (SP)**	3.19	.82	0.32	0.36	0.46	0.40	0.46	(.87)	
**Self-deception (SD)**	.97	1.36	0.47	0.54	0.50	0.42	0.49	0.43	(.74)

Study C N = 330. All dimensions modeled as reflective factors (5 items each) and means and standard deviations are based on average of items scores for each factor, except self-deception which is recoded to account for extreme responses and calculated as a sum.

**Table 5:** QMCA Validity and Reliability Assessments.

	Convergent Validity			Discriminant Validity			Reliability Decomposition	
	AVE	CR	α	Max R	Loading range	Δ AVE to MSV	Substantive Reliability loading	Method Reliability loading (%)
**Self-Awareness**	.38	.75	.74	.49	.50 −.74	.12	.67	.09 (12%)
**Self-Control**	.42	.78	.78	.67	.54 −.76	.12	.67	.11 (14%)
**Adaptability**	.55	.86	.82	.58	.61 −.78	.21	.65	.18 (21%)
**Achievement Orientation**	.55	.86	.89	.58	.75 −.82	.21	.75	.14 (16%)
**Teamwork**	.56	.86	.82	.51	.62 −.77	.30	.64	.19 (22%)

Study C N = 330. Estimates based on CFA modeled with method factor controls (self-promotion and self-deception). AVE = Average Variance Extracted; CR = Composite Reliability; α = Cronbach’s alpha; Max *R* = Maximum Factor Correlations; MSV = Maximum shared variance; ASV = Average Shared Variance. Reliability Decomposition calculated based on the Method-U mode.

### Self-report measurement bias assessment

We used the CFA marker technique [61–63] to assess the effect of impression management bias on the QMCA measures (Table 6). This method was not applied to the self-deception measure as it was formulated as a composite variable to capture extreme responses. The CFA marker technique evaluates changes in model fit across different models to detect method bias. In the baseline model (Model B), impression management loadings were set based on the original measurement model, with the method factor uncorrelated with substantive factors. In the constrained model (Model C), items for the substantive factors were allowed to load to impression management with equal loadings, while in the unconstrained model (Model U), these loadings were freely estimated. Factor correlation effects were tested in Model R which built on the best-fitting model (C or U) by constraining correlations between the substantive factors to their values in Model B.

**Table 6:** QMCA Model Fit Comparisons for Impression Management Effects.

Overall Model Fit	χ^2^	df	*p*	CFI
1.CFA	763.172	390	<.001	.91
2.Baseline	853.260	404	<.001	.90
3.Method-C	755.599	403	<.001	.92
4.Method-U	701.133	379	<.001	.93
5.Method-R	710.984	389	<.001	.93
**Model Fit Comparisons**	**Δ χ** ^**2**^	**Δ df**	***p***	**Δ CFI**
Baseline vs Method-C	97.661	1	<.001	−.02
Method-C vs Method-U	54.466	24	<.001	−.01
Method R vs Method-U	9.851	10	.454	0

Study C N = 330. CFA model: Original measurement model with no constraints. Baseline Model: Impression management loadings fixed and constrained to not correlate with competency factors. Method C Model: Competency items allowed to load equally on Impression Management factor to reflect method variance. Method U Model: Method variance loadings allowed to vary across items. Method R Model: Correlations between competency factors constrained to values in Baseline Model.

Results confirmed that participants’ assessments of their competencies were biased by impression management as indicated by a significant decline in model fit from Model B to Model C. Model fit significantly improved when method effects were freely estimated (Model U), suggesting that impression management effects varied across items. However, decomposed reliability demonstrated that all factors remained reliable after accounting for such bias (composite reliability range:.64−.75, see Table 5). Additionally, model comparisons showed that controlling for impression management did not alter factor correlations. The results suggest that convergent and discriminant validity of the QMCA scale is robust after accounting for the impression management effects on participant self-reports.

### Measurement invariance

Research suggests that non-cognitive assessments do not demonstrate differences based on gender or race [9–11,64]. To assess whether the QMCA meets this criterion, we tested multigroup invariance across gender and race/ethnicity using the Vandenberg and Lance confirmatory factor analysis method [65]. For gender, we compared the individuals identifying as male and female, representing 98.2% of the original sample (N = 324). For race/ethnicity, due to small subgroup sizes, we compared the individuals identifying as white to those identifying as a race or ethnicity other than white, representing 97.9% of the original sample (N = 323). Models were estimated in MPLUS using maximum likelihood with Satorra-Bentler corrections (MLM) which is robust to non-normality.

Configural models showed good fit, indicating that factor structure and the pattern of loadings is equivalent across groups (Gender: CFI = .92, RMSEA = .05, p-close = .25; Race/Ethnicity: CFI = .92, RMSEA = .05, p-close = .42). Based on nonsignificant difference in the Satorra-Bentler Chi-Square statistic for the nested models and decline in the CFI of less than.01 [66]. Metric invariance was supported, demonstrating consistent relationships between items and constructs across groups (Gender: scaled ∆S-Bχ^2^ [25]=19.26, p = .79, ∆CFI = −.002; Race/Ethnicity: scaled ∆S-Bχ^2^ = 25.98, p = .41, ∆CFI = −.001). The QMCA achieved full scalar invariance for gender (scaled ∆S-Bχ^2^ [51]=65.57, p = .07, ∆CFI = −.005) and partial scaler invariance for race/ethnicity with one item in the teamwork factor allowed to vary (scaled ∆S-Bχ^2^ [50]=63.82, p = .08, ∆CFI = −.005), indicating that item intercepts and thus the scaling of item responses were predominantly equivalent across groups. Recent work suggests that relaxing the strict assumption that all items are equivalent such as through partial invariance supports meaningful and valid group comparisons [67,68].

We then tested latent mean differences by freely estimating means in one group. For gender, no significant differences were found for adaptability (∆Mean = −.01, p = .97), achievement orientation (∆Mean = .18, p = .16) or teamwork (∆Mean = .11, p = .39). Women reported higher self-awareness (Cohen’s d = .38, p < .05), while men reported higher self-control (Cohen’s d = −.36, p < .01). For race/ethnicity, no significant differences emerged for any factors (Self-awareness: ∆Mean = −.06, p = .66; Self-control: ∆Mean = .07, p = .58; Adaptability: ∆Mean = −.13, p = .29; Achievement Orientation: ∆Mean = −.04, p = .73; Teamwork: ∆Mean = .05, p = .69). Table 7 sets forth model fit and model fit difference tests for all models.

**Table 7:** QMCA Measurement Invariance Results.

	Overall Model Fit					Model Fit Comparison				
**Gender**	**S-B χ** ^**2**^	**df**	**p**	**CFI**	**RMSEA**		**∆S-B χ** ^**2**^	**∆df**	**p**	**∆CFI**
1. Configural	773.05	530	<.001	.917	.053					
2. Metric	794.578	555	<.001	.919	.052	1–2	19.26	25	.79	−.002
3. Scalar	839.506	580	<.001	.912	.053	1–3	65.57	50	.07	−.005
**Race/ Ethnicity**	**S-B χ2**	**df**	**p**	**CFI**	**RMSEA**		**∆S-B χ** ^**2**^	**df**	**p**	**∆CFI**
1. Configural	753.311	530	<.001	.922	.051					
2. Metric	781.051	555	<.001	.921	.050	1–2	25.98	25	.41	−.001
3. Partial Scalar	818.011	579	<.001	.917	.051	1–3	63.82	49	.08	−.005

Gender Invariance N = 324; Race/Ethnicity Invariance N = 323. Based on 25-item, 5-factor model of substantive competencies. S-B χ^2^ = Satorra-Bentler adjusted chi-square statistic; CFI = Comparative Fit Index; RMSEA = Root mean square error of approximation; **∆**S-B χ^2^ = scaled chi-square difference of nested model^74^.

Our preliminary invariance testing suggests that the QMCA provides an impartial tool to assess competencies across groups. The scale demonstrated strong invariance at the configural, metric and scalar levels for gender and race/ethnicity. While our sample showed differences in self-awareness and self-control across gender, they had small effect sizes. Future research should explore whether those differences persist in other samples and whether they influence on substantive relationships [69] with outcomes such as school retention and success. A key limitation of our analysis is that it relies solely on self-report responses. Since the QMCA can also be used for 360-degree rating processes that incorporate external evaluations, future studies should assess invariance in that context. Additionally, our sample size constraints limited the ability to examine invariance and latent mean differences across more specific gender and racial/ethnic subgroups, which may also be explored in future work.

## Discussion

While holistic admissions practices hold great promise for helping to diversify graduate education, the reality of the typical admissions process is that diversity, if considered at all, is often a parameter used after the applicant pool has been filtered by factors that have disparate impact on women and minority applicants [1,2]. The QMCA was an equivalent measure of competencies across race and gender for all measures and did not demonstrate differences in competency ratings across race and only small differences in two factors, self-awareness and self-control across gender. Thus, the using the QMCA as a complement to cognitive assessments early in the admissions process may avoid excluding of members of underrepresented groups who have the potential to excel in graduate STEMM studies in the selection process. Research suggests that unlike standardized testing, there is no score gap with noncognitive assessments [70]. While data is limited to this point on the correlation of ESI and academic success for minority groups, one study with Black/African American students showed a positive correlation between academic achievement and the ability to recognize, use, and manage emotions. This suggests that ESI skills may help mitigate the effects of systemic barriers and enhance academic outcomes for underrepresented groups [71].

For diversity-friendly practices to be adopted by university staff and faculty, they must be easy to use, like GPA and GRE scores. The QMCA serves this need for ESI competencies.

In 2023, the QMCA was augmented to enable collection of 360-degree rater data to complement the self-report data from the students. This enables the QMCA to provide a holistic picture of how the participant views their own behavior, as well as others’ perceptions of that individual’s enacted behaviors. The 360-degree assessment, also known as multi-source feedback, is a process where individuals receive confidential, anonymous feedback from the people who work with them. In organizational contexts, this typically includes the employee’s manager, peers, and direct reports. However, the 360-method has also been used extensively with student groups; where students seek feedback from colleagues, professors, friends, and family. A variety of feedback from multiple sources provides a well-rounded view of an individual’s performance, behaviors, and interactions within the organization [72]. Providing a validated, easy to use tool that produces a simple report of self- or both self- and 360-degree ratings will make it easier for institutions to incorporate the QMCA as part of the admissions process. It is argued that this comprehensive approach facilitates a more nuanced understanding of behaviors, skills, and areas for improvement, thereby enhancing the validity and reliability of research findings [73]. In addition to quantitative evaluations, the report may also include verbatim comments from the raters that help reviewers and participants understand the context behind the scores, aligning with the intent of holistic admissions processes.

The QMCA may also have applications beyond admissions, particularly in student development and mentoring. Prior research suggests that feedback on EI and SI competencies can support student self-awareness and professional growth. By incorporating the QMCA into coaching and mentoring programs for students, institutions may be able to provide targeted developmental opportunities. However, further research is needed to determine whether using the QMCA in these contexts leads to measurable improvements in student persistence and success rates.

The verbatim feedback included in the QMCA reports may be particularly helpful in development to enable students to understand the context behind their scores. Implementing these assessments in admissions and student support programs can help promote a more equitable and inclusive educational environment, leading to increased access and improved retention rates for underrepresented groups. The validation of the QMCA represents a step toward integrating structured assessments of EI and SI into STEMM graduate education. While the QMCA provides a reliable and valid measure of key EI and SI competencies, its broader implications for admissions, retention, and student success remain open questions for future research. Further studies should explore how QMCA results correlate with academic and professional outcomes and whether its use in graduate admissions and mentoring programs contributes to more inclusive and supportive STEMM environments.

Through extensive field-testing and data analysis, five factors emerged within our model (self-awareness, self-control, adaptability, achievement orientation, and teamwork). The QMCA is weighted toward assessing emotional competencies related to understanding and managing oneself, with a single social competency (teamwork) included to address relation management skills. Future studies could expand the QMCA to include more social competencies related to developing and sustaining healthy interpersonal relationships. For example, empathy and cultural awareness can make for better teachers and working environments by enhancing inclusivity.

Thus, users can triangulate results of the QMCA by employing both self and other ratings to assess behavioral competencies related to emotional intelligence. We acknowledge that certain ESI competencies, such as self-awareness and self-management may be less transparent to third parties. As these competencies may be best measured using self-reports, we have also developed method bias controls designed to target the specific self-report biases of impression management and self-decision that are common concerns when assessing ESI. Our validation study suggests that the method factors improve the accuracy of the self-assessments, but future research is needed to confirm that the method factors are controlling bias and not accounting for another substantive trait. Another method to triangulate results would be to supplement the Likert-scale questions with situational judgement and scenario-based questions that can yield a more comprehensive understanding of ESI competency levels. Combining multiple question forms would enable assessment of not only the individual’s displayed competencies through their actions/behavior, but also their grasp of emotions and how effectively they apply this understanding.

Medical education also stands to gain from such assessments. There is a growing body of research on emotional and social competencies in healthcare related domains, including medical education, practice and training, nursing, and performance improvement interventions [46]. However, there remains a need for further investigation. By assessing how students approach situations, even if they lack certain skills, context-based scenarios and situational judgement questions can provide insights into areas where students may need further training or development. In healthcare, these assessments offer a comprehensive view of student capabilities, enabling trainers to deliver targeted feedback and effectively guide skill development.

We developed the QMCA to create a resource to identify and support potential graduate student applicants from underrepresented groups who may be overlooked by traditional admissions methods. Thus, the QMCA focuses on ESI competencies which do not systematically vary across demographic groups [9–11,64] as do traditional measures of cognitive intelligence, such as the standardized tests. Although the competencies were selected to reduce systematic differentiation due to majority and minority status, an important next step is to assess measurement invariance of the QMCA. Invariance testing with small samples sizes leads to highly unreliable results, accordingly, we could not test invariance in our validation study (Study C). Future studies should seek to assess the QMCA on samples with sufficiently large and balanced group membership in categories such as gender, race and ethnicity, nationality, first generation college students, to ensure systematic group differences do not significantly affect measurement of the competencies.

Finally, one of the fundamental criticisms of EI is the lack of consensus on what it measures. Different researchers have proposed various models, leading to confusion and difficulty in studying EI as a coherent concept. For instance, Salovey and Mayer, who originally coined the term, define EI in terms of the ability to monitor one’s own and others’ feelings and emotions, to discriminate among them, and to use this information to guide one’s thinking and actions [74]. In contrast, Goleman expanded the concept to include a wide range of skills and traits, such as self-regulation, motivation, and social skills [33]. This discrepancy in definitions complicates the measurement and application of EI [74]. Some argue that much of the research on EI lacks rigorous experimental design, making it difficult to establish causal relationships between EI and outcomes like job performance, academic success, or leadership effectiveness [75]. However, new applied research is continuous and while these concerns remain, new measures, such as the QMCA are needed to continue rigorous research on emotional intelligence.

### Implications for curriculum and pedagogy

Emotional and social competency training included in academic programs prepare students for both academic challenges and future scientific professions. Programs like the SELECT MD Program at the Morsani College of Medicine at the University of South Florida have integrated a comprehensive focus on emotional and social competencies to augment the traditional core undergraduate medical school (MD) curriculum [76]. Programs like SELECT provide a roadmap for schools that may wish to include a dedicated focus to the development of emotional and social competencies.

### Barriers and considerations

The integration of emotional and social competencies within natural sciences faces hurdles. As pointed out by Bennett and Gadlin [77], there exists a traditional emphasis on cognitive skills in natural sciences which sometimes overshadows the importance of emotional and social aspects. The term “emotional intelligence” has been met with challenges, despite its growing popularity over the past decades. To overcome this, institutions need to actively engage faculty and students, highlighting the critical role of emotional intelligence in personal and professional success, citing the numerous studies that support the impact of ESI. Research shows that higher emotional intelligence is linked to better academic performance, improved mental health, and enhanced leadership abilities [20,74,78]. This effort is essential for gaining the necessary buy-in and fully integrating emotional intelligence into educational programs.

The number of studies within the field remains small, and further research should be done as differences (or lack thereof) can depend on the measurement model of emotional intelligence used, as well as the specific populations and contexts studied. Additional studies with larger samples of URM and more women are needed.

Some critics have highlighted potential cultural bias in EI assessments and training programs. They argue that what is considered emotionally intelligent behavior can vary widely across different cultures, and therefore standardized EI measures may not be universally applicable or fair. However, within a professional context, the behaviors that are measured through the QMCA and other similar behavioral competency assessments provide an accurate assessment of the behaviors as agreed upon norms, and which are necessary for individual and organizational success.

When using emotional and social competencies as criteria, it is important to recognize that other individual differences may impact results. For example, neurodiversity, cultural background, and other life experiences may influence an individual’s emotional and social intelligence profile. Similarly, certain mental health conditions might influence emotional regulation and social functioning, affecting assessment results. Therefore, it is crucial to consider assessments in the context and allow for holistic assessments of individuals. While cognitive ability is a strong predictor of job performance, it is not sufficient on its own. Other factors, such as personality traits and emotional intelligence, also play critical roles in predicting individual and organizational outcomes [79]. The QMCA is not intended as a standalone measure, but one used in conjunction with other measures to provide a more holistic understanding than personality and cognitive capabilities can alone. A follow up study is recommended utilizing longitudinal data on student performance.

The QMCA should have broad usage outside of natural sciences, yet to this point the sample data has been collected from that specific population. Further study in applying this tool to other roles and industries is needed with data collection occurring within a diverse population. However, similar assessments of behavioral competencies, such as the ESCI, has had wide data collection and validation across roles, industries, and geographic areas [80].

## Supporting information

S1 Appendix QMCA Scale and Scoring. (DOCX)
