# Supplementary material for: Development of a survey tool to assess emotional and social behavioral competencies of science technology engineering math and medicine (STEMM) graduate students
Source: PLoS One. 2025 Sep 2;20(9):e0328308. doi: 10.1371/journal.pone.0328308 (PMC12404483; doi:10.1371/journal.pone.0328308)
Supplement: S1 Appendix — (DOCX) [file pone.0328308.s001.docx]

**Appendix A. QMCA Scale and Scoring**

**QMCA ESI Competencies**

| Self-Awareness | 1 | I recognize how my feelings affect what I say and do. |
| --- | --- | --- |
|  | 2 | I notice when things affect how I feel. |
|  | 3 | I notice when others impact how I feel. |
|  | 4 | I notice how I am feeling. |
|  | 5 | I recognize the reasons for my feelings. |
| Self-Control | 1 | I pause before I react in stressful situations. |
|  | 2 | I calmly deal with challenges when they arise. |
|  | 3 | I resist acting on impulse. |
|  | 4 | I maintain my composure in difficult situations. |
|  | 5 | I take steps to manage my mood if something upsets me. |
| Adaptability | 1 | I adapt my plans when situations change. |
|  | 2 | I use a variety of resources or approaches to get things done. |
|  | 3 | I approach things in different ways if ’hey don't work the first time. |
|  | 4 | I change how I do things to get results. |
|  | 5 | In new situations, I adapt quickly. |
| Achievement Orientation | 1 | I strive to achieve excellence in my work. |
|  | 2 | I seek to improve my abilities. |
|  | 3 | I look for ways to improve my performance. |
|  | 4 | I challenge myself to accomplish goals. |
|  | 5 | I strive to do my best. |
| Teamwork | 1 | I like working with others toward a common goal. |
|  | 2 | In group projects, I seek out roles that require cooperating with others. |
|  | 3 | When working with a group, I seek out input from others before taking action. |
|  | 4 | I incorporate ideas from others when working in a team. |
|  | 5 | In group projects, I consider the needs of my team members. |

All items measured on a 5-point Likert Scale. 1=”Never” 5 = “Consistently”

**Social Desirability Controls**

| Self-Promotion^16^ | 1 | I speak proudly about my experience or education. |
| --- | --- | --- |
|  | 2 | I make others aware of my talents and qualifications. |
|  | 3 | I let others know that I am valuable to the group. |
|  | 4 | I let others know when I have a reputation for being competent in a particular area. |
|  | 5 | I make people aware of my accomplishments. |
| Self-deception | 1 | Rational thinking drives my decision-making. |
|  | 2 | I correctly predict how I will respond in challenging situations. |
|  | 3 | I accurately assess my own ability as compared to my peers. |
|  | 4 | I correctly predict the consequences of my actions. |
|  | 5 | I accurately anticipate how others feel. |

All items measured on a 5-point Likert Scale. 1=”Never” 5 = “Consistently”

**Scoring Instructions.**

Competency scores are independent of each other and should not be aggregated. We outline 2 methods to estimate the competency values for students.

- Method 1 – Averaging. This provides simple scores for each competency and levels of socially desirable responding for each individual. These scores are not adjusted for measurement error or socially desirable responding.
- Method 2 – Factor Analysis. This provides factor scores for each competency which are adjusted for measurement error and levels of self-promotion and self-deception.

**Method 1 – Estimating Simple Scores:**

**Competency Estimates:** To estimate simple scores for each competency, average the item values for each competency.

**Social Desirability Estimates:** The self-promotion score is estimated the same way as the other competencies, by averaging the item scores. The self-deception measure is estimated by assigning 1 point to each item that is rated a 5 (“Consistently” and aggregating the values, thus scores will range from 0 to 5. This scoring system is similar to how other self-deception measures are scored (51, 80).

**Method 2 – Estimating Simple Factor Scores**

Use confirmatory factor analysis to estimate the model including all competencies and self-promotion and the composite Self-deception variable. Load the relevant items to their referent factor for all competencies and self-promotion (e.g., all self-awareness items should be loaded to the self-awareness factor). The Self-deception variable is calculated the same way it is in Method 1. Allow all factors to correlate with each other and with the self-deception variable. Save the factor scores which represent the level of each competency adjusted for measurement error and level of social desirability.

You may also assess the performance of the self-promotion measure as a method marker using the CFA marker method proposed by Williams, Hartman and Cavazotte (67).
